# Supplementary material for: Development of a computer-aided design software for the quantitative evaluation of aesthetic damage
Source: PLoS One. 2019 Dec 18;14(12):e0226322. doi: 10.1371/journal.pone.0226322 (PMC6919621; doi:10.1371/journal.pone.0226322)
Supplement: S1 Code site — 1. (ZIP) [file pone.0226322.s001.zip › [EESC_jr][Projeto]Relatorio_07_21/reports/templates_zip/gentelella-master/vendors/select2/docs/examples.html]

---
layout: default
title: Examples - Select2
slug: examples
---

# Examples

{% include examples/basics.html %}
{% include examples/placeholders.html %}
{% include examples/data.html %}
{% include examples/disabled-mode.html %}
{% include examples/disabled-results.html %}
{% include examples/multiple-max.html %}
{% include examples/hide-search.html %}
{% include examples/programmatic-control.html %}
{% include examples/tags.html %}
{% include examples/tokenizer.html %}
{% include examples/matcher.html %}
{% include examples/localization-rtl-diacritics.html %}
{% include examples/themes-templating-responsive-design.html %}

{% include nav/examples.html %}

{% include js-source-states.html %}
